# Interleukin-1β Polymorphisms Are Genetic Markers of Susceptibility to Periprosthetic Joint Infection in Total Hip and Knee Arthroplasty

**DOI:** 10.3390/genes15050596

**Published:** 2024-05-08

**Authors:** Valentina Granata, Dario Strina, Valentina Possetti, Roberto Leone, Sonia Valentino, Katia Chiappetta, Mattia Loppini, Alberto Mantovani, Barbara Bottazzi, Rosanna Asselta, Cristina Sobacchi, Antonio Inforzato

**Affiliations:** 1 IRCCS Humanitas Research Hospital, Rozzano, 20089 Milan, Italy; valentina.granata@humanitasresearch.it (V.G.); dario.strina@humanitasresearch.it (D.S.); valentina.possetti@humanitasresearch.it (V.P.); roberto.leone@humanitasresearch.it (R.L.); sonia.valentino@humanitasresearch.it (S.V.); katia.chiappetta@humanitas.it (K.C.); mattia.loppini@hunimed.eu (M.L.); alberto.mantovani@humanitasresearch.it (A.M.); barbara.bottazzi@humanitasresearch.it (B.B.); rosanna.asselta@hunimed.eu (R.A.); cristina.sobacchi@humanitasresearch.it (C.S.); 2 Milan Unit, Institute for Genetic and Biomedical Research, National Research Council, 20138 Milan, Italy; 3 Department of Biomedical Sciences, Humanitas University, 20072 Pieve Emanuele, Italy; 4 Fondazione Livio Sciutto Onlus, Campus Savona, Università degli Studi di Genova, 16126 Savona, Italy; 5 Harvey Research Institute, Queen Mary University of London Charterhouse Square, London EC1M 6BQ, UK

**Keywords:** periprosthetic infection, single-nucleotide polymorphism, association analysis, haplotype, epistasis, biomarker, PTX3

## Abstract

Periprosthetic joint infections (PJIs) are serious complications of prosthetic surgery. The criteria for the diagnosis of PJI integrate clinical and laboratory findings in a complex and sometimes inconclusive workflow. Host immune factors hold potential as diagnostic biomarkers in bone and joint infections. We reported that the humoral pattern-recognition molecule long pentraxin 3 (PTX3) predicts PJI in total hip and knee arthroplasty (THA and TKA, respectively). If and how genetic variation in *PTX3* and inflammatory genes that affect its expression (*IL-1β*, *IL-6*, *IL-10*, and *IL-17A*) contributes to the risk of PJI is unknown. We conducted a case–control study on a Caucasian historic cohort of THA and TKA patients who had prosthesis explant due to PJI (cases) or aseptic complications (controls). Saliva was collected from 93 subjects and used to extract DNA and genotype *PTX3*, *IL-1β*, *IL-6*, *IL-10*, and *IL-17A* single-nucleotide polymorphisms (SNPs). Moreover, the concentration of IL-1β, IL-10, and IL-6 was measured in synovial fluid and plasma. No association was found between *PTX3* polymorphisms and PJI; however, the AGG haplotype, encompassing rs2853550, rs1143634, and rs1143627 in *IL-1β*, was linked to the infection (*p* = 0.017). Also, synovial levels of all inflammatory markers were higher in cases than in controls, and a correlation emerged between synovial concentration of PTX3 and that of IL-1β in cases only (Spearman r = 0.67, *p* = 0.004). We identified a relationship between rs2853550 and the synovial concentration of IL-1β and PTX3. Our findings suggest that *IL-1β* SNPs could be used for the early identification of THA and TKA patients with a high risk of infection.

## 1. Introduction

Periprosthetic joint infections (PJIs) are amongst the most devastating and frequent complications of prosthetic surgery. Mainly caused by coagulase- and Gram-positive *Staphylococcus aureus* (*S. aureus*) and coagulase- and Gram-negative *S. epidermidis*, these infections require aggressive antimicrobial treatments; however, their efficacy is hampered by the structure of the bone, making it an elusive target for systemic drugs and often resulting in revision surgery [1]. Despite a decrease in the incidence of PJI over the last few decades, these infections still cause failure in 15–25% of total hip or knee arthroplasties (THA and TKA, respectively) [2]. Moreover, the number of these orthopedic procedures is ever-growing due to the aging of the population, with projected rises of 174% in THA and 673% in TKA by 2030 [3]. In an era of emerging antibiotic resistance, this picture points to PJI as a highly impacting disease in terms of morbidity, social and economic costs, sustainability for the healthcare systems, and, ultimately, mortality (with rates of 5–8%) [4].

In 2018, the European Bone and Joint Infection Society (EBJIS) elaborated a definition of PJI that integrates clinical and laboratory findings, including evaluation of inflammatory markers in the synovial compartment (i.e., white blood cell count, leukocyte esterase, and histology of synovial tissue) and in the blood (i.e., erythrocyte sedimentation rate (ESR), C-reactive protein (CRP), and interleukin 6 (IL-6)), imaging (i.e., X-rays, bone scan, magnetic resonance imaging (MRI), computed tomography (CT), and positron emission tomography (PET)), and microbiology (i.e., Gram stain, bacterial cultures, and matrix-assisted laser desorption ionization–time of flight mass spectrometry) [5,6]. These guidelines have been recently revised [7], yet the diagnostic workflow remains complex, time consuming, and inconclusive in some cases. Furthermore, the relationship between host genetics and the risk of PJI is overlooked in the clinical management of the disease.

In recent years, several studies have been conducted to identify novel biomarkers that aid in the diagnosis of PJI in the pre-operative stage (i.e., prior to revision surgery) [8]. In a prospective diagnostic study, Deirmengian and colleagues found that the concentration in the synovial fluid of antimicrobial factors, such as α-defensin, neutrophil elastase (ELA-2), bactericidal permeability-increasing protein (BPI), lactoferrin, and neutrophil gelatinase-associated lipocalin (NGAL), increased in PJI patients, thus suggesting that these factors are biomarkers of the infection [9]. Indeed, α-defensin has been incorporated in the most recent EBJIS definition of confirmed PJI [7]. We recently reported that the synovial levels of the long pentraxin 3 (PTX3) predict PJI with high specificity (93.33%) in a cohort of THA and TKA patients that were eligible for revision surgery [10]. Locally synthesized and released at sites of inflammatory and infectious insult both by immune and non-immune cells, PTX3 is a soluble pattern-recognition molecule (PRM) that acts as an early indicator of tissue damage and remodeling [11]. Clear correlations have emerged in numerous studies between systemic (i.e., in the plasma) and local (i.e., in the bronchoalveolar lavage (BAL)) levels of PTX3 and both the presence and severity of infections caused by diverse pathogens (e.g., *Aspergillus fumigatus*-*A. fumigatus*, *Pseudomonas* species, and, more recently, SARS-CoV-2) [12,13,14], and an established body of evidence points to this PRM as a diagnostic/prognostic marker of opportunistic infections, including those sustained by *S. aureus* [11]. Of note, PTX3 is acknowledged as a component of the bone microenvironment (BME), whereby human mesenchymal and osteoblast (OB) cells express the protein, and this promotes OB differentiation and mineral matrix deposition [15,16]. Also, PTX3 has been associated with metabolic, autoimmune, and inflammatory diseases of the skeleton [17].

Three common single-nucleotide polymorphisms (SNPs) in the *PTX3* gene (namely, rs2305619, rs3816527, and rs1840680) have been associated with the concentration of the PTX3 protein in biological fluids (i.e., plasma and BAL [18,19]), the risk of opportunistic infections of bacterial [12,20,21] and fungal [19,22,23] etiology, and the severity of COVID-19 disease [24]. Also, the transcription of *PTX3* has been reported to be controlled by two enhancers whose methylation status changes in inflammatory conditions; interestingly, one of them encompasses or is proximal to the three SNPs indicated above, which might explain why these polymorphisms affect the expression of the protein. In addition to epigenetic mechanisms, PTX3 expression is well known to be induced both by pro- and anti-inflammatory cytokines, including IL-1β and IL-10 [11,25]. In this regard, IL-10 synergizes with Toll-like receptor (TLR) agonists (e.g., lipopolysaccharides (LPSs)) and IL-1β to promote the synthesis and release of PTX3 by myeloid cells (primary cellular players in PJI pathogenesis), thus pointing to this pentraxin as an important modulator of inflammation (e.g., in the context of bone infections) [25]. Interestingly, SNPs in the genes coding for IL-1β and IL-10 and other mediators that crosstalk to these cytokines and are key components of the inflammatory milieu of the infected bone (i.e., *IL-17A* and *IL-6* [26]) have been associated with osteoarticular infections [27,28,29,30]. In this regard, the rs1143627 SNP in the *IL-1β* gene has been linked to the risk of post-traumatic osteomyelitis in a Chinese Han cohort [27], and polymorphisms in the *IL-10* gene have been proposed as genetic risk factors for chronic periodontitis [29] and hematogenous osteomyelitis [30]. Also, synovial IL-6 has been documented to detect PJI in a cohort of THA and TKA patients [31], and the rs1800796 polymorphism in the *IL-6* gene has been associated with post-traumatic osteomyelitis [27]. Moreover, polymorphisms in the *IL-17A* gene have been reported to affect the protein’s expression, and an association has been observed between synovial and serum levels of IL-17A and PJI [28]. This information notwithstanding, the combined contribution of these genetic factors to the risk of PJI in THA and TKA patients is not clear.

With the general aim of identifying novel genetic signatures of PJI, we therefore conducted a case–control retrospective study on an historic cohort of patients that underwent revision of THA/TKA at the Department of Orthopaedics and Trauma Surgery of IRCCS Humanitas Research Hospital and participated in a previous investigation [10]. The primary endpoint of the study was to assess the relationship between the risk of PJI and genotypes/haplotypes of polymorphisms in the *PTX3*, *IL-6*, *IL1-β*, *IL-17A*, and *IL-10* genes that were selected based on prior knowledge. As a secondary endpoint, we evaluated the relationship between these genetic variants and the concentration of the corresponding proteins in the synovial fluid and in the plasma, with a focus on PTX3, which we recently proposed as a novel marker of PJI. We envisage that the genetic and biochemical signatures identified in our study could be exploited in the clinic for risk stratification of THA and TKA patients and optimization of the criteria currently used to diagnose PJI.

## 2. Materials and Methods

### 2.1. Study Design and Population Size

We conducted a retrospective case–control study on a historic cohort of unrelated subjects of Caucasian origin enrolled in a previous investigation in our Hospital (registration number: 165/2017; see [10]). These were THA/TKA patients who underwent revision surgery and met the following inclusion criteria: painful THA or TKA for at least 3 months, and sufficient clinical and laboratory data to define the presence or absence of PJI. Exclusion criteria were the use of antibiotics, glucocorticoids, and anti-histaminic therapy in the 2 weeks prior to surgery; rheumatoid arthritis and other rheumatic disorders; revision surgery for spacer removal and reimplantation; metallosis; prosthetic dislocation; periprosthetic fracture; limb length discrepancy; prosthetic rupture; and polyethylene wear. Revisions were classified as septic (cases, hereafter infected) or aseptic (controls, hereafter non-infected) according to the 2018 EBJIS criteria [32]. Based on these criteria, an increased synovial fluid leukocyte count was defined as a leukocyte count of >2000/mL or >70% granulocytes, positive histopathology was defined as a mean of >23 granulocytes per 10 high-power fields (HPFs), confirmatory microbial growth in synovial fluid and periprosthetic tissue cultures was considered positive if 1 specimen was positive for highly virulent organisms (e.g., *S. aureus*) or 2 specimens were positive for low virulent pathogens, and sonication culture was considered positive if >50 colony-forming units (CFU)/mL of sonicated fluid were counted [32]. To evaluate the relationship between gene polymorphisms and PJI, patients were enrolled also who lacked synovial fluid [10]. The presentation of the study and collection of signed informed consent were carried out by trained and qualified physicians of the Department of Orthopaedics and Trauma Surgery of IRCCS Humanitas Research Hospital from March 2021 to December 2023. In total, 93 subjects were enrolled from the original cohort of which 46 had PJI and 47 had aseptic prosthesis revision. Samples of saliva were collected from these patients that were used to extract genomic DNA and genotype selected SNPs, as detailed below. Samples of synovial fluid and plasma (stored at −80 °C) were available from the previous study [10]. Clinical data were accessed through the electronic record of the hospital. The study complied with the provisions of the Declaration of Helsinki and was approved by the Institutional Review Board of IRCCS Humanitas Research Hospital (registration number: 2835/2021). Data generated in the study (with major regard to the genetic information) were processed, analyzed, and stored in compliance with current Legislation.

### 2.2. Collection and Processing of Saliva

Samples of saliva were collected using the GeneFix^TM^ Saliva DNA Collection Device (Isohelix^TM^, Cell Projects Ltd., Harrietsham, UK), which contains a solution that stabilizes nucleic acid, allowing for long-term storage at room temperature, and inactivates microorganisms, including enveloped viruses (e.g., SARS-CoV-2). DNA was extracted from salivary cells using the MaxWell^TM^ AS1000 system (Promega, Madison, WI, USA), following the manufacturer’s instructions. Conditioned to quality and quantity check on a NanoDrop spectrophotometer (Thermofisher, Waltham, MA, USA), DNA samples were kept at 4 °C until use.

### 2.3. SNP Genotyping

All SNPs analyzed in the study are listed in Table 1. In detail, the three common SNPs in the *PTX3* gene (namely rs2305619, rs3816527, and rs1840680) were genotyped by PCR amplification of genomic DNA, followed by Sanger sequencing (primer sequences are available upon request); this strategy allowed us to annotate the genotypes of 11 additional rare SNPs encompassed in the amplicons. Polymorphisms in the *IL-1β*, *IL-6*, *IL-10,* and *IL-17A* genes (selected based on prior knowledge; see Section 1) were genotyped using commercial Taqman probes with 2 different dyes (VIC™ and FAM™) for the detection of either allele on a ViiA7 System (Applied Biosystem, Waltham, MA, USA), following the manufacturer’s protocol.

### 2.4. Quantitation of Inflammatory Markers

The concentrations of IL-1β, IL-10, IL-6, and CRP (a PTX3 homolog extensively used in the clinic as a non-specific marker of inflammation [33]) in both synovial fluid and, for IL-10, plasma were quantified using the Ella™ Automated Immunoassay System (Bio-Techne, Minneapolis, MN, USA), following the manufacturer’s instructions. IL-17A was not quantitated.

The levels of PTX3 in the synovial fluid and plasma, as well as those of clinically established plasmatic markers (erythrocyte sedimentation rate, ESR, CRP, and D-Dimer) were from our previous study [10]. In all cases, half LOD (limit of detection) values were arbitrarily assigned to samples whose readings were below the LOQ (limit of quantitation).

### 2.5. Statistical Analysis

Genetic data were analyzed using the open-source PLINK software, v. 1.07 (http://zzz.bwh.harvard.edu/plink/, accessed on 18 January 2024) [34]. Prior to performing association tests, we checked each polymorphism for possible deviation from the Hardy–Weinberg equilibrium --hardy command of the PLINK tool). We then assessed the association of alleles, genotypes, and haplotypes of the selected polymorphisms both with disease (PJI) and concentration of proteins in the synovial fluid and, when available, plasma. For each SNP, a standard case–control analysis with chi-square or R^2^ test was run to estimate the odds ratio (ORs) or β indices (depending on whether the outcome variable was binary or continuous) with the corresponding *p*-values and 95% confidence interval (CI), taking the minor allele as a reference. Haplotype analysis and phasing were performed using the sliding-window method. Associations with *p* < 0.05 were taken as statistically significant unless otherwise stated (i.e., considering Bonferroni correction for multiple testing). Power estimates indicated that, if each analyzed polymorphism (allele frequency of 10 or 20%) was to directly confer a 2-fold increase in the relative risk of infection, the case and control groups used in this research would be of sufficient size to have only 31 or 40% power to detect a significant association at the 0.05.

Graphs and plots were generated using Graph-Pad Prism v. 9.5 (GraphPad, Boston, MA, USA). Continuous variables were assessed for normality; outliers were identified using the ROUT routine of Prism (with a Q of 1%) and excluded from graphs and statistical testing.

## 3. Results

### 3.1. Study Population

**Figure 1:**
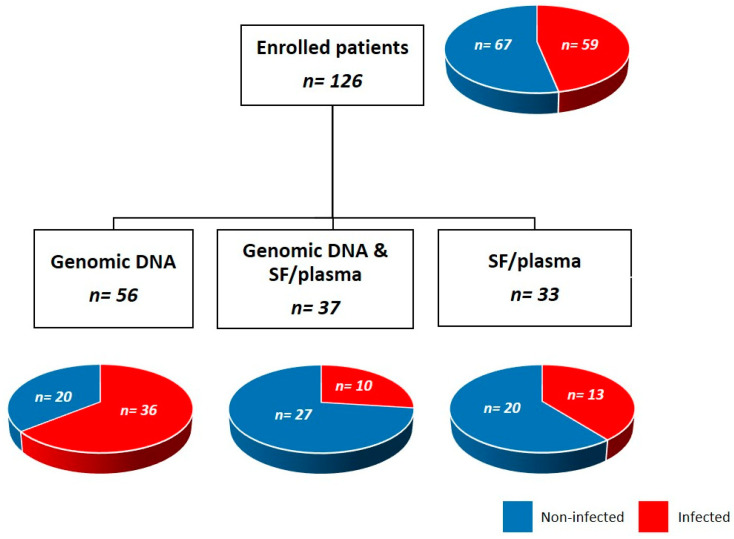
Breakdown of the study population based on the biological materials analyzed in the study (SF, synovial fluid).

We conducted genetic association analyses on a sub-cohort (126 subjects) of THA/TKA patients who had been enrolled in our previous study [10]. At the time of enrolment, these patients had undergone revision surgery at the Department of Orthopaedics and Trauma Surgery of IRCCS Humanitas Research Hospital and met the inclusion/exclusion criteria listed in the Section 2 for the diagnosis of PJI [32]. Based on disease status, this historic cohort was therefore retrospectively divided into two groups, i.e., infected (cases) and non-infected (controls) patients. Of the 126 subjects who participated in the present study, 93 subjects, comprising 46 cases and 47 controls, donated a sample of saliva that was used to isolate genomic DNA and perform the genetic analyses described below. Also, samples of synovial fluid and plasma collected in our previous study were used to quantitate IL-1β, IL-6, CRP (in the synovial fluid only), and IL-10 (both in the synovial fluid and plasma). In more detail, for 37 of the 93 genotyped patients (27 infected and 10 non-infected), samples of synovial fluid and plasma were available, whereas the remaining 56 (20 infected and 36 non-infected) had none. In addition, 33 patients (20 infected and 13 non-infected) were enrolled that provided samples of synovial fluid and plasma only (see Figure 1 for a schematic of the study population).

Cases and controls were comparable in terms of gender, body mass index (BMI), age at surgery, and Charlson Comorbidity Index (CCI) (see Table 2). Also, the non-infected group had a larger proportion of cases with mild systemic disease (score = 2, 61.2% vs. 39% in Table 2), based on the American Society of Anesthesiologists (ASA) score.

### 3.2. Microbiology and Inflammatory Markers

The bacteria of the *Staphylococcus* genus were the most frequent microbial isolates in the study cohort (found in 23 out of 59 infected patients), with a prevalence in the THA subgroup (see Table 3). Twelve patients were positive for *S. epidermidis*; five for *S. aureus*; and six for other *Staphylococcus* species, including *S. lugdunensis*, *S. caprae*, and *S. capitis*. The presence of other pathogens was rare, irrespective of surgery (i.e., THA or TKA). Also, the relative proportions of coagulase-negative (e.g., *S. epidermidis*), coagulase-positive (e.g., *S. aureus*), and Streptococci species were consistent with those observed in our previous study [10].

The concentration of PTX3 in the synovial fluid and plasma, the concentrations of CRP and D-Dimer in the plasma, and the ESR values were retrieved from our records [10]. In the present study, the levels of synovial PTX3, plasmatic CRP, and ESR were higher in the cases than the controls, while plasmatic PTX3 and D-Dimer did not differ between the two groups (Table 4). This is coherent with the findings from our past investigations, except for ESR that did not change with the disease status in the parental cohort [10]. Also, the synovial concentration of PTX3 was higher in patients with diagnosis of PJI even when the study population was stratified based on the explanted implant (THA or TKA, as previously reported [10].

**Figure 2:**
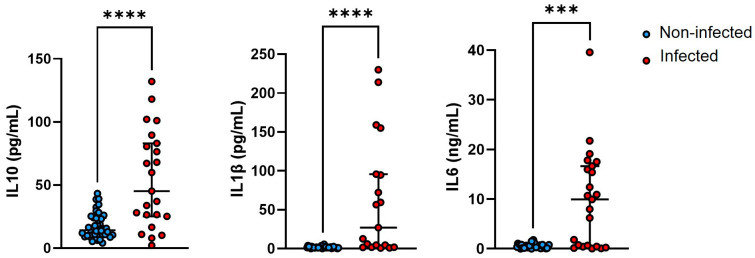
Concentration of inflammatory mediators in the synovial fluid. The synovial levels of IL-10, IL-1β, and IL-6 (pg/mL or ng/mL) were measured using the Ella™ Automated Immunoassay System. Each circle in the plots corresponds to a patient (either infected in red, or non-infected in blue). Outlier-free data are shown that were obtained from 70 patients. Mann–Whitney test, *** *p* < 0.001, **** *p* < 0.0001.

The concentration of IL-1β, IL-10, IL-6, and CRP in the synovial fluid (and, for IL-10, in the plasma too) was measured in 70 patients (that had both biological fluids available, see Figure 1), using high-sensitivity commercial immunoassays (Ella™). As shown in Figure 2, infected patients had higher concentrations of synovial IL-10 (*p* < 0.0001), IL-1β (*p* < 0.0001), and IL-6 (*p* < 0.001). Also, the levels of CRP in the synovial fluid were increased in the cases compared to the controls (*p* < 0.0001, Supplementary Figure S1a), in agreement with the trend observed for this marker in the plasma (Table 4). As opposed to this, the plasmatic concentration of IL-10 was not affected by the disease status (Supplementary Figure S1b), consistent with the view that this cytokine is synthesized and secreted locally (at sites of infection), as proposed for PTX3 [10].

### 3.3. Gene Polymorphisms and Risk of PJI

The samples of saliva collected in the study were used to extract genomic DNA from salivary cells. The extracted DNA was either PCR-amplified (to Sanger sequence the *PTX3* gene) or directly applied to genotyping assays based on commercial Taqman probes (designed and validated for the selected polymorphisms).

Of the 14 SNPs encompassed in the *PTX3* amplicons (listed in Table 1), 10 were found to be monomorphic, and 1 (rs35948036) was rare (2 out of 93 genotyped individuals), in agreement with the frequency data available for these variants from the Genome Aggregation Database (gnomAD v4.0.0, https://gnomad.broadinstitute.org/, accessed on 11 March 2024; the reported minor allele frequency, MAF, for these SNPs ranges from 0.003 to 0.03). The remaining three SNPs (rs2305619, rs3816527, and rs1840680) were present in both allelic forms in our cohort, consistent with their frequency estimates in gnomAD (MAF in a European population > 0.4). These SNPs are localized in different regions of the *PTX3* gene: rs3816527 (A > C) is in the coding region (c.143) and causes an amino acid substitution (Ala > Asp) at position 48 of the preprotein, while rs2305619 (A > G) and rs1840680 (A > G) are intronic variants (c.130 + 9 and c.472 + 326, respectively). They have been previously associated with susceptibility to microbial infections [12,19,20,21,22,23,24]; in our cohort, their allelic frequencies were similar in infected (F_A) and not-infected (F_U) patients (Table 5). The allelic frequencies of polymorphisms in the *IL-6* and *IL-17A* genes were not linked to PJI either. However, our data suggested that the intronic SNPs rs3024491 in *IL-10* and rs2853550 in *IL-1β* had different allelic frequencies in cases and controls (Table 5). Indeed, the rs3024491-A allele had a trend of higher frequency in non-infected compared to infected patients (51.09% vs. 32.95%, *p* = 0.01), and rs2853550-A was more frequent in cases than in controls (15.56% vs. 4.35%, *p* = 0.01; Table 5). It could be argued that, in our cohort of THA and TKA patients, the rs3024491-A variant in *IL-10* was associated with a lower probability of PJI (OR = 0.47; 95%CI = 0.26–0.86), while the rs2853550-A variant in *IL-1β* was associated with a higher probability of infection (OR = 4.05; 95%CI = 1.28–12.93).

For all tested SNPs, no significant deviation from the Hardy–Weinberg equilibrium was observed either in the cases or in the controls (or in both).

We performed genotype-based association analyses by focusing on polymorphisms that had different allelic frequencies in infected and non-infected patients, i.e., *IL-10* and *IL-1β*. As shown in Table 6, genetic testing for dominant and recessive inheritance suggested that the rs3024491-A and rs2853550-A variants in the *IL-10* and *IL-1β* genes, respectively, both exerted dominant effects on the genotypic association with PJI, consistent with the allelic distributions shown in Table 5.

After seeing these findings, we evaluated the relationship between PJI and haplotypes formed by the polymorphisms in *IL-10* and *IL-1β*. Interestingly, the haplotype-association analyses indicated that the AGG haplotype (contributed by the rs2853550, rs1143634, and rs1143627 variants in *IL-1β*) was approximately four times more frequent in infected than in non-infected individuals (12.86% vs. 3.22%, *p* = 0.017) and strongly associated with PJI (Table 7). As opposed to this, IL-10 haplotypes had no clear link with the infection. The allelic, genotypic, and haplotypic relationships here described were all retained when patients’ characteristics (i.e., age, sex, and BMI) were modeled into the analysis (i.e., in a multivariate setting.

### 3.4. Relationship between Gene Polymorphisms and Concentration of Inflammatory Markers

We recently documented that the synovial levels of PTX3 are strongly associated with PJI in THA and TKA patients that underwent revision surgery [10,11]. IL-10, IL-1β, and IL-6 have also been proposed as synovial markers of bone infections [32,33,34,35], and their concentration was much increased in the synovial fluid of the infected subjects of our cohort (see Table 4 and Figure 2). Interestingly, polymorphisms in the *PTX3*, *IL-10*, *IL-1β*, and *IL-6* genes are known to be associated with the level of the corresponding proteins in biological fluids. For example, the concentration of PTX3 in the BAL of bone marrow-transplanted patients with a diagnosis of invasive pulmonary aspergillosis has been reported to change depending on haplotypes encompassing the rs2305619, rs3816527, and rs1840680 SNPs [19]. Similar relationships have been found in the plasma of healthy individuals [18]. We therefore assessed the association between polymorphisms and the synovial concentration of the proteins by taking patients’ characteristics (age, sex, and BMI) and disease status (infected, non-infected) as covariates in the applied PLINK models. Our genotype-based analyses indicated that the rs1143634 SNP in *IL-1β* had a trend of association with the IL-1β concentration in the synovial fluid (*p* = 0.04; Table 8), with no further links emerging from other genes and proteins. Also, we extended these investigations to the relationship between SNPs in the *PTX3* and *IL-10* genes and concentration of the corresponding proteins in the plasma; however no effect of these allelic variants could be observed at a systemic level.

PTX3 expression is induced in several immune and non-immune cells, including monocytes, osteoblasts, endothelial, epithelial, and dendritic cells, by inflammatory cytokines (in addition to intact microorganisms and microbial moieties), with IL-1β acting as a primary trigger [11,16,25]. We therefore evaluated the relationships between polymorphisms in the *IL-1β* gene and synovial concentration of the PTX3 protein. We found that the rs2853550 SNP in the *IL-1β* gene was strongly associated with the levels of this soluble PRM in the synovial fluid (Table 9), suggesting that gene variability in *IL-1β* affects the expression of PTX3, possibly through intermediate effects on the IL-1β protein (Table 8).

**Figure 3:**
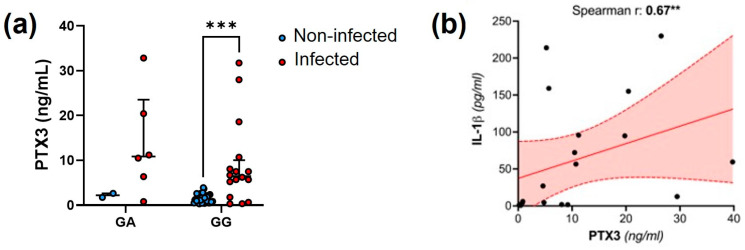
Genetic and biochemical associations between PTX3 and IL-1β. (**a**) Distribution of the PTX3 synovial concentration across infected and non-infected patients carrying either the GA or GG genotype of the *IL-1β* SNP rs2853550. The AA data are not shown because only 1 patient (infected) had this genotype. Two-way ANOVA, *** *p* < 0.001. (**b**) Spearman correlation between synovial levels of IL-1β and PTX3. The red area delimited by the dotted lines indicates the 95%CI, and the solid line is the linear regression curve. ** *p* < 0.01.

These relationships were corroborated by the observation that the synovial concentration of PTX3 was higher in individuals with PJI that were homozygous for the G variant of the rs2853550 polymorphism in the IL-1β gene than in those carrying alternative genotypes (Figure 3a). Also, a strong and positive correlation emerged between the synovial levels of IL-1β and PTX3 (Spearman r = 0.67, *p* = 0.004; Figure 3b) that was lost in the absence of infection.

Collectively, these findings strongly suggest a relationship between the production of IL-1β at sites of infection, the local induction of PTX3, and the underlying genetic variability.

## 4. Discussion

THA and TKA are orthopedic procedures performed with ever-growing frequency owing to population aging, and they bear the risk of PJI; this, in turn, may lead to severe healthcare and socio-economic burden [35]. The treatment of these infections often relies on the administration of massive doses of antibiotics; however, this may be insufficient, resulting in surgical revision of prosthesis. Nonetheless, the infection may persist and evolve towards antimicrobial resistance, thus forcing patients into a (sometime life-long) therapy odyssey. The identification of novel specific and highly sensitive biomarkers would allow for a more rapid and accurate diagnosis and treatment of PJIs, possibly improving patient outcomes [36]. In particular, the identification of genetic variability (e.g., SNPs) associated with susceptibility to PJIs might allow for the establishment timely preventative measures. An association with primary (i.e., less than 3 months after surgery) or revision PJI has been suggested so far for polymorphisms in diverse genes, including *IL-1β*, *IL-6*, Tumor Necrosis Factor-α (*TNF-α*), Colony Stimulating Factor 3 Receptor (*CSF3R*), Interleukin-1 receptor antagonist (*IL-1 RN*), Mannose Binding Lectin (*MBL*)-1 and -2, and Vitamin D Receptor (*VDR*) [37,38]. SNPs in many other genes, such as those encoding matrix metalloproteinases (MMPs), Receptor Activator of NF-kB (RANK), RANK ligand, and Osteoprotegerin (OPG), have been tested, but they failed to show significant associations [38]. However, there is a lack of confirmatory studies in the field.

Here, we genotyped selected variants of the genes coding for the long pentraxin PTX3 and inflammatory cytokines (IL1-β, IL-6, IL-10, and IL-17A) in a cohort of THA/TKA patients and assessed the relationships between genotype, allele, and haplotype frequencies and both disease status and proteins’ level. In our study cohort, we did not observe significant associations between genetic variants in *PTX3* and PJI. In fact, only 3 out of the 14 SNPs genotyped in *PTX3* (rs2305619, rs3816527, and rs1840680) were polymorphic, however with no difference in terms of frequency across infected and non-infected patients. This is likely due to the limited number of patients enrolled in the study; based on our experience with PTX3 genetics and susceptibility to opportunistic infections [11], we envisage that follow-up investigations on a larger population would be powered enough to detect significant associations with the risk of infection.

As opposed to the polymorphisms in *PTX3*, the rs3024491 in *IL-10* and rs2853550 in *IL-1β* were strongly associated with the infection status. These polymorphisms are already associated with the risk of bacterial infections [39]. In our study, the rs3024491-A allele was more frequent in patients who had aseptic prosthesis revision and had a dominant pattern of inheritance. Importantly, in patients with prosthesis infection, the minor allele (A) of the rs2853550 SNP in the *IL-1β* gene was more frequent than the alternative one (G). Also, the AGG haplotype in this gene had a strong association with the infection, indicating that, in our cohort, carriers of the rs2853550-A variant had a higher risk of PJI. The rs2853550 SNP was previously reported to be associated with bone diseases, including infections [27] and ankylosing spondylitis [40].

**Figure 4:**
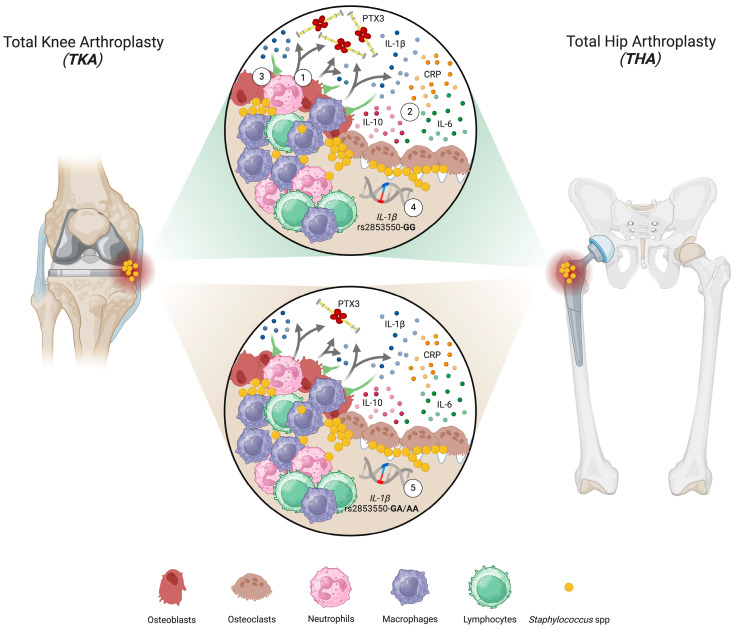
Gene and protein crosstalk between PTX3 and IL-1β in PJI. Pathogenic microorganisms, including *S. aureus* (SA), colonize the bone microenvironment (BME) through hematogenous spreading or contiguous infections. Pathogen-associated molecular patterns (PAMPs) on bone-seeded SA induce bone-resident cells (mostly osteoblasts) and immune cells (i.e., neutrophils, macrophages, and lymphocytes) (1; grey arrows) to release inflammatory mediators, including IL-1β, IL-10, IL-6, CRP, and PTX3, via pattern-recognition receptors (PRRs) (2). Locally released IL-1β further stimulates the synthesis and secretion of PTX3 (3; green lines). Variability in the *IL-1β* gene is associated with expression of PTX3, possibly due to intermediate effects on production of IL-1β. In this regard, the synovial concentration of PTX3 was higher in individuals with PJI that were homozygous for the G variant of the rs2853550 polymorphism in the *IL-1β* gene (4) than in those carrying alternative genotypes (5). Created with BioRender.com accessed on 4 April 2024.

We recently proposed PTX3 as a sensitive and specific synovial marker of PJI [10]. Here, we observed that the synovial concentrations of IL-1β, IL-10, and IL-6 also were increased in infected patients. High levels of synovial IL-1β have been documented, especially in cases of *S. aureus*-dependent PJI [41]. Similarly, synovial IL-10 has been found to increase during PJIs, particularly in chronic condition [27]. Also, IL-6 has been proposed as a biomarker for the diagnosis of PJI [42] and non-infectious complications of dental implants. For example, higher IL-6 concentrations have been detected in the peri-implant crevicular fluid of not-infected patients carrying dental implants and presenting peri-implantitis as compared to individuals with peri-mucositis or showing no clinical signs of inflammation [43]. Interestingly, the synovial levels of PTX3 in our cohort of infected patients were strongly associated with gene variability in *IL-1β*. This association was lost in non-infected patients who had reduced levels of the IL-1β protein. In this regard, this cytokine is known to be a major stimulus for PTX3 expression in immune and non-immune cells, including myeloid dendritic cells [25] and trophoblasts [44]. Consistent with this, the synovial concentration of PTX3 significantly correlated with that of IL-1β. Also, IL-10 has been reported to synergize with IL-1β in promoting PTX3 expression [25]; however, we did not see any effect of gene variability in *IL-10* on the synovial levels of the PTX3 protein. Based on these findings, we envisage a relationship between the gene variability in *IL-1β*, production of this cytokine at sites of infection, and induction of PTX3 that might be of clinical relevance to improve the power of PTX3 (and, possibly, IL-1β) in the diagnosis of PJI in THA and TKA patients, as detailed in Figure 4. 

In conclusion, our clinical study identified a panel of genetic markers associated with infection in a relatively small cohort of patients with PJI. The validation of the associations here described in a larger cohort and the confirmation of the prognostic value of this genetic signature with respect to susceptibility to PJI in a prospective study could pave the way to genetic-driven targeted preventive treatments, eventually relieving the burden posed by these insidious infections.

## Figures and Tables

**Table 1:** Genes and SNPs analyzed in the study.

Gene	SNP	Position	Gene	SNP	Position
*PTX3*	rs75123224	Chr3:157436911	*PTX3*	rs16827644	Chr3:157442520
	rs35948036	Chr3:157436979		rs35415718	Chr3:157442702
	rs138898586	Chr3:157436987		rs138818541	Chr3:157442912
	rs34655398	Chr3:157437050	*IL-1* *β*	rs1143627	Chr2:112836810
	rs2305619	Chr3:157437072		rs1143634	Chr2:112832813
	rs3816527	Chr3:157437525		rs2853550	Chr2:112829544
	rs148943471	Chr3:157437630	*IL-6*	rs1800797	Chr7:22726602
	rs112277608	Chr3:157437858		rs1800796	Chr7:22726627
	rs191352729	Chr3:157437882	*IL-10*	rs3024491	Chr1:206771701
	rs199772915	Chr3:157437913		rs1800872	Chr1:206773062
	rs1840680	Chr3:157438240	*IL-17A*	rs2275913	Chr6:52186235

**Table 2:** Characteristics of the study population.

Characteristic	All Patients (*n* = 126)	Infected (*n* = 59)	Non-Infected (*n* = 67)	*P*
Sex (M:F)	62:64	34:25	28:39	0.08 ^b^
BMI ^a^	28.0 (24.6–30.2)	29.4 (25.4–32.0)	26.8 (24.7–29.5)	0.10 ^c^
CCI	0: 7 pts (5.6%)	0: 5 pts (8.5%)	0: 2 pts (3%)	0.29 ^b^
	1: 16 pts (12.7%)	1: 8 pts (13.6%)	1: 8 pts (12%)	
	2: 35 pts (27.8%)	2: 19 pts (32.2%)	2: 16 pts (24%)	
	3: 33 pts (26.2%)	3: 10 pts (17%)	3: 23 pts (34.3%)	
	4: 16 pts (12.7%)	4: 7 pts (11.9%)	4: 9 pts (13.4%)	
	5: 9 pts (7.1%)	5: 3 pts (5.0%)	5: 6 pts (9%)	
	6: 3 pts (2.4%)	6: 2 pts (3.4%)	6: 1 pts (1.5%)	
ASA score	1: 27 pts (21.4%)	1: 17 pts (28.8%)	1: 10 pts (15%)	0.05 ^b^
	2: 64 pts (50.8%)	2: 23 pts (39%)	2: 41 pts (61.2%)	
	3: 28 pts (22.2%)	3: 14 pts (23.7%)	3: 14 pts (20.9%)	
Age at surgery ^a^	68 (60–75)	70 (61.5–76)	66 (57–74)	0.17 ^c^

^a^ Age in years; median and IQR. ^b^ Chi-square. ^c^ Mann–Whitney. BMI, body mass index. M, male; F, female. CCI, Charlson Comorbidity Index. ASA, American Society of Anesthesiologists. CCI and ASA score were not available for 7 patients, including 5 infected and 2 non-infected subjects.

**Table 3:** Pathogens identified at the time of THA/TKA revision surgery.

Pathogen	N (Hip:Knee)	Pathogen	N (Hip:Knee)
*S. epidermidis*	12 (11:1)	*Enterococcus faecalis*	2 (1:1)
*S. aureus*	5 (4:1)	*E. faecium*	2 (1:1)
Other *Staphylococcus* spp.	6 (6:0)	*Corynebacterium* spp.	1 (0:1)
*Streptococcus sanguinis*	1 (0:1)	*Propionibacterium acnes*	2 (1:1)
Other *Streptococcus* spp.	3 (3:0)	*A. fumigatus*	1 (1:0)
*Brevibacillus laterosporus*	1 (0:1)	*Salmonella* spp.	1 (0:1)
*Bacillus* spp.	2 (2:0)	*Ralstonia pickettii*	1 (1:0)
*Pseudomonas aeruginosa*	2 (2:0)	Not identified	17 (10:7)

**Table 4:** Concentration of inflammatory markers in the synovial fluid (PTX3) and in the plasma.

Marker	All Patients (*n* = 126)	Infected (*n* = 59)	Non-Infected (*n* = 67)	*p*
Synovial PTX3 (ng/mL)	2.4 (0.9–8.6)	8.7 (4.8–22)	1.8 (0.8–2.6)	<0.0001
Plasmatic PTX3 (ng/mL)	5.3 (3.7–6.5)	5.0 (3.3–6.5)	5.3 (4.0–6.7)	0.2561
Plasmatic CRP (mg/dL)	0.5 (0.2–1.0)	0.8 (0.3–1.8)	0.3 (0.09–0.6)	<0.0001
Plasmatic ESR (mm/hr)	20.0 (9–37)	23.5 (12.5–47.8)	15.0 (8.0–28.3)	0.0082
Plasmatic D-Dimer (ng/mL)	293 (246.5–374.5)	288 (247.3–388)	308 (236–376)	0.840

Values are indicated as median and IQR. *p*-values are from the Mann–Whitney test.

**Table 5:** Distribution of allelic frequencies in the *PTX3*, *IL-10*, *IL-1β*, *IL-6,* and *IL-17A* SNPs across infected (affected, A; *n* = 46) and non-infected (unaffected, U; *n* = 47) patients.

Gene	SNP	A1	F_A	F_U	A2	χ^2^	*p*	OR	L95	U95
*IL-10*	rs3024491	A	32.95	51.09	C	6.06	**0.01**	0.47	0.26	0.86
	rs1800872	T	38.64	30.43	G	1.34	0.24	1.44	0.78	2.67
*IL-1β*	rs2853550	A	15.56	4.348	G	6.41	**0.01**	4.05	1.28	12.83
	rs1143634	A	26.67	21.11	G	0.76	0.38	1.36	0.68	2.71
	rs1143627	G	34.44	25	A	1.94	0.16	1.58	0.83	2.30
*PTX3*	rs2305619	A	41.49	45.56	G	0.31	0.58	0.85	0.47	1.52
	rs3816527	C	38.30	38.64	A	0.00	0.96	0.99	0.54	1.79
	rs1840680	A	41.49	47.78	G	0.74	0.39	0.78	0.43	1.39
*IL-17A*	rs2275913	G	32.05	37.5	A	0.52	0.47	0.79	0.41	1.52
*IL-6*	rs1800797	A	37.21	26.67	G	2.25	0.13	1.63	0.86	3.09
	rs1800796	C	11.11	17.39	G	1.46	0.23	0.59	0.25	1.39

F_A, frequency in affected; F_U, frequency in unaffected; χ^2^, chi-square test; OR, odds ratio; L95 and U95, low and upper 95% confidence interval; *p*, *p*-value. Suggestive *p*-values are in bold (Bonferroni *p*-value threshold = 0.0045).

**Table 6:** Distribution of genotypes in the *IL-10* and *IL-1β* SNPs across infected (affected, A; *n* = 46) and non-infected (unaffected, U; *n* = 47) patients.

Gene	SNP	A1	A2	Test	A	U	χ^2^	*p*
*IL-10*	rs3024491	A	C	GENO	7/15/22	11/25/10	7.85	**0.019**
		A	C	DOM	22/22	36/10	7.84	**0.0051**
		A	C	REC	7/37	11/35	0.90	0.34
*IL-1β*	rs2853550	A	G	GENO	2/10/33	0/4/42	5.64	0.06
		A	G	DOM	12/33	4/42	5.07	**0.024**
		A	G	REC	2/43	0/46	2.09	0.15

A, affected; U, unaffected; χ^2^, chi-square test; *p*, *p*-value. GENO, genotypic test of association in the 2-by-3 table of disease-by-genotype; DOM, genetic test for a dominant model of inheritance of the minor allele; REC, genetic test for a recessive model of inheritance of the minor allele. Suggestive *p*-values are in bold.

**Table 7:** Distribution of the haplotypes formed by the *IL-10* and *IL-1β* polymorphisms across infected (affected, A; *n* = 46) and non-infected (unaffected, U; *n* = 47) patients.

Gene	Haplotype	F_A	F_U	χ^2^	*p*
*IL-10*	OMNIBUS	NA	NA	7.22	0.065
	AT	2.99	8.23	2.27	0.14
	CT	34.22	22.20	3.18	0.074
	AG	29.57	42.86	3.39	0.066
	CG	33.22	26.71	0.90	0.34
*IL-1β*	OMNIBUS	NA	NA	9.86	0.079
	GAG	4.80	1.18	2.06	0.15
	AGG	12.86	3.22	5.74	**0.017**
	GGG	16.79	20.59	0.43	0.51
	GAA	21.87	20.13	0.08	0.77
	AGA	2.70	1.11	0.60	0.44
	GGA	40.99	53.74	2.97	0.085

F_A, frequency in affected; F_U, frequency in unaffected; χ^2^, chi-square test; *p*, *p*-value; NA, not applicable. Significant *p*-values are in bold and red (Bonferroni *p*-value threshold = 0.025).

**Table 8:** Analysis of the genotypic association between polymorphisms in the *PTX3*, *IL-10*, *IL-1β*, and *IL-6* genes and proteins’ concentration in the synovial fluid.

Relationship	SNP	β	Stat	*p*
*PTX3* SNPs ↔ [PTX3]	rs2305619	−0.24	−0.15	0.88
	rs3816527	0.29	0.18	0.86
	rs1840680	0.65	0.43	0.67
*IL-10* SNPs ↔ [IL-10]	rs3024491	−2.63	−0.53	0.60
	rs1800872	−1.09	−0.22	0.82
*IL-1β* SNPs ↔ [IL-1β]	rs2853550	−2775	−0.04	0.97
	rs1143634	−91.90	−2.17	**0.041**
	rs1143627	25.74	0.72	0.48
*IL-6* SNPs ↔ [IL-6]	rs1800797	−671.60	−13	0.22
	rs1800796	−1136	−1.72	0.10
	rs1800796	−1136	−1.72	0.10

β, probability index; STAT, statistical testing; *p*, *p*-value. Suggestive *p*-values are in bold (Bonferroni *p*-value threshold = 0.017).

**Table 9:** Analysis of the genotypic association between polymorphisms in the *IL-1β* gene and the synovial concentration of PTX3.

Relationship	SNP	β	Stat	*p*
*IL-1β* SNPs ↔ [PTX3]	rs2853550	9.33	3.95	**0.00024**
	rs1143634	−1.18	−0.59	0.56
	rs1143627	0.99	0.51	0.61

β, probability index; SD: standard deviation; STAT, statistical testing; *p*, *p*-value. Significant *p*-values are in bold and red (Bonferroni *p*-value threshold, *p* = 0.017).

## Data Availability

The original data presented in the study are openly available in IRCCS Humanitas Research Hospital & Humanitas University at https://zenodo.org/communities/humanitasirccs (accessed on 14 April 2024).

## Supplementary Materials

The following supporting information can be downloaded at https://www.mdpi.com/article/10.3390/genes15050596/s1, Figure S1: Synovial CRP and plasma IL-10 concentration.

## References

[1] Patel R. (2023). Periprosthetic Joint Infection. N. Engl. J. Med..

[2] Bozic K.J., Kurtz S.M., Lau E., Ong K., Chiu V., Vail T.P., Rubash H.E., Berry D.J. (2010). The epidemiology of revision total knee arthroplasty in the United States. Clin. Orthop. Relat. Res..

[3] Kurtz S., Ong K., Lau E., Mowat F., Halpern M. (2007). Projections of primary and revision hip and knee arthroplasty in the United States from 2005 to 2030. J. Bone Jt. Surg. Am..

[4] Fischbacher A., Borens O. (2019). Prosthetic-joint Infections: Mortality over the last 10 years. J. Bone Jt. Infect..

[5] Leone S., Borre S., d’Arminio Monforte A., Mordente G., Petrosillo N., Signore A., Venditti M., Viale P., Nicastri E., Lauria F.N. (2010). Consensus document on controversial issues in the diagnosis and treatment of prosthetic joint infections. Int. J. Infect. Dis..

[6] Signore A., Sconfienza L.M., Borens O., Glaudemans A.W.J.M., Cassar-Pullicino V., Trampuz A., Winkler H., Gheysens O., Vanhoenacker F.M.H.M., Petrosillo N. (2019). Consensus document for the diagnosis of prosthetic joint infections: A joint paper by the EANM, EBJIS, and ESR (with ESCMID endorsement). Eur. J. Nucl. Med. Mol. Imaging.

[7] McNally M., Sousa R., Wouthuyzen-Bakker M., Chen A.F., Soriano A., Vogely H.C., Clauss M., Higuera C.A., Trebše R. (2021). The EBJIS definition of periprosthetic joint infection: A practical guide for clinicians. Bone Jt. J..

[8] Jacovides C.L., Parvizi J., Adeli B., Jung K.A. (2011). Molecular markers for diagnosis of periprosthetic joint infection. J. Arthroplast..

[9] Deirmengian C., Kardos K., Kilmartin P., Cameron A., Schiller K., Parvizi J. (2019). Diagnosing Periprosthetic Joint Infection: Has the Era of the Biomarker Arrived?. Clin. Orthop. Relat. Res..

[10] Loppini M., Di Maio M., Avigni R., Leone R., Inforzato A., Grappiolo G., Mantovani A., Bottazzi B. (2023). Long Pentraxin 3 as a New Biomarker for Diagnosis of Hip and Knee Periprosthetic Joint Infections. J. Clin. Med..

[11] Garlanda C., Bottazzi B., Magrini E., Inforzato A., Mantovani A. (2018). PTX3, a Humoral Pattern Recognition Molecule, in Innate Immunity, Tissue Repair, and Cancer. Physiol. Rev..

[12] Jaillon S., Moalli F., Ragnarsdottir B., Bonavita E., Puthia M., Riva F., Barbati E., Nebuloni M., Cvetko Krajinovic L., Markotic A. (2014). The humoral pattern recognition molecule PTX3 is a key component of innate immunity against urinary tract infection. Immunity.

[13] Mauri T., Coppadoro A., Bombino M., Bellani G., Zambelli V., Fornari C., Berra L., Bittner E.A., Schmidt U., Sironi M. (2014). Alveolar pentraxin 3 as an early marker of microbiologically confirmed pneumonia: A threshold-finding prospective observational study. Crit. Care.

[14] Lapadula G., Leone R., Bernasconi D.P., Biondi A., Rossi E., D’Angiò M., Bottazzi B., Bettini L.R., Beretta I., Garlanda C. (2022). Long pentraxin 3 (PTX3) levels predict death, intubation and thrombotic events among hospitalized patients with COVID-19. Front. Immunol..

[15] Grčević D., Sironi M., Valentino S., Deban L., Cvija H., Inforzato A., Kovačić N., Katavić V., Kelava T., Kalajzić I. (2018). The long pentraxin PTX3 plays a role in bone turnover and repair. Front. Immunol..

[16] Parente R., Sobacchi C., Bottazzi B., Mantovani A., Grčević D., Inforzato A. (2019). The Long Pentraxin PTX3 in Bone Homeostasis and Pathology. Front. Immunol..

[17] Tarantino U., Greggi C., Cariati I., Visconti V.V., Gasparini M., Cateni M., Gasbarra E., Botta A., Salustri A., Scimeca M. (2021). The Role of PTX3 in Mineralization Processes and Aging-Related Bone Diseases. Front. Immunol..

[18] Barbati E., Specchia C., Villella M., Rossi M.L., Barlera S., Bottazzi B., Crociati L., d’Arienzo C., Fanelli R., Garlanda C. (2012). Influence of Pentraxin 3 (PTX3) Genetic Variants on Myocardial Infarction Risk and PTX3 Plasma Levels. PLoS ONE.

[19] Cunha C., Aversa F., Lacerda J.F., Busca A., Kurzai O., Grube M., Löffler J., Maertens J.A., Bell A.S., Inforzato A. (2014). Genetic deficiency of PTX3 and aspergillosis in stem cell transplantation. N. Engl. J. Med..

[20] Chiarini M., Sabelli C., Melotti P., Garlanda C., Savoldi G., Mazza C., Padoan R., Plebani A., Mantovani A., Notarangelo L.D. (2010). PTX3 genetic variations affect the risk of *Pseudomonas aeruginosa* airway colonization in cystic fibrosis patients. Genes Immun..

[21] Olesen R., Wejse C., Velez D.R., Bisseye C., Sodemann M., Aaby P., Rabna P., Worwui A., Chapman H., Diatta M. (2007). DC-SIGN (CD209), pentraxin 3 and vitamin D receptor gene variants associate with pulmonary tuberculosis risk in West Africans. Genes Immun..

[22] He Q., Li H., Rui Y., Liu L., He B., Shi Y., Su X. (2018). Pentraxin 3 Gene Polymorphisms and Pulmonary Aspergillosis in Chronic Obstructive Pulmonary Disease Patients. Clin. Infect. Dis..

[23] Tang T., Dai Y., Zeng Q., Bu S., Huang B., Xiao Y., Wei Z., Lin X., Huang L., Jiang S. (2020). Pentraxin-3 polymorphisms and pulmonary fungal disease in non-neutropenic patients. Ann. Transl. Med..

[24] Feitosa T.A., de Souza Sá M.V., Pereira V.C., de Andrade Cavalcante M.K., Pereira V.R.A., da Costa Armstrong A., do Carmo R.F. (2023). Association of polymorphisms in long pentraxin 3 and its plasma levels with COVID-19 severity. Clin. Exp. Med..

[25] Doni A., Michela M., Bottazzi B., Peri G., Valentino S., Polentarutti N., Garlanda C., Mantovani A. (2006). Regulation of PTX3, a key component of humoral innate immunity in human dendritic cells: Stimulation by IL-10 and inhibition by IFN-γ. J. Leukoc. Biol..

[26] Granata V., Possetti V., Parente R., Bottazzi B., Inforzato A., Sobacchi C. (2022). The osteoblast secretome in *Staphylococcus aureus* osteomyelitis. Front. Immunol..

[27] Jiang N., Li S.Y., Ma Y.F., Hu Y.J., Lin Q.R., Yu B. (2020). Associations between Interleukin Gene Polymorphisms and Risks of Developing Extremity Posttraumatic Osteomyelitis in Chinese Han Population. Mediat. Inflamm..

[28] Erdemli B., Özbek E.A., Başarir K., Karahan Z.C., Öcal D., Biriken D. (2018). Proinflammatory biomarkers’ level and functional genetic polymorphisms in periprosthetic joint infection. Acta Orthop. Traumatol. Turc..

[29] Moudi B., Heidari Z., Mahmoudzadeh-Sagheb H., Moudi M. (2018). Analysis of interleukin-10 gene polymorphisms in patients with chronic periodontitis and healthy controls. Dent. Res. J..

[30] Osman A.E., Mubasher M., ElSheikh N.E., AlHarthi H., AlAlallah I.A., Elbeshir A.A., Abashar M., Elsidig N., ElGhazali G., Fadil A.S. (2015). Investigation of polymorphisms in anti-inflammatory cytokine genes in hematogenous osteomyelitis. Genet. Mol. Res..

[31] Randau T.M., Friedrich M.J., Wimmer M.D., Reichert B., Kuberra D., Stoffel-Wagner B., Limmer A., Wirtz D.C., Gravius S. (2014). Interleukin-6 in serum and in synovial fluid enhances the differentiation between periprosthetic joint infection and aseptic loosening. PLoS ONE.

[32] Li C., Renz N., Trampuz A. (2018). Management of Periprosthetic Joint Infection. Hip Pelvis..

[33] Kushner I. (2023). C-reactive protein—My perspective on its first half century, 1930–1982. Front. Immunol..

[34] Purcell S., Neale B., Todd-Brown K., Thomas L., Ferreira M.A., Bender D., Maller J., Sklar P., de Bakker P.I., Daly M.J. (2007). PLINK: A tool set for whole-genome association and population-based linkage analyses. Am. J. Hum. Genet..

[35] Zardi E.M., Franceschi F. (2020). Prosthetic joint infection. A relevant public health issue. J. Infect. Public Health.

[36] Schindler M., Nike W., Maderbacherm G., Sigmund I.K., Alt V., Rupp M. (2023). Novel diagnostic markers for periprosthetic joint infection: A systematic review. Front. Cell. Infect. Microbiol..

[37] Zhou X., Yishake M., Li J., Jiang L., Wu L., Liu R., Xu N. (2015). Genetic susceptibility to prosthetic joint infection following total joint arthroplasty: A systematic review. Gene.

[38] Hijazi A., Hasan A., Pearl A., Memon R., Debeau M., Roldan M., Awad M.E., Abdul-Kabir E., Saleh K.J. (2022). Genetic Polymorphisms Associated with Perioperative Joint Infection following Total Joint Arthroplasty: A Systematic Review and Meta-Analysis. Antibiotics.

[39] Assis S., Marques C.R., Silva T.M., Costa R.S., Alcantara-Neves N.M., Barreto M.L., Barnes K.C., Figueiredo C.A. (2014). IL10 single nucleotide polymorphisms are related to upregulation of constitutive IL-10 production and susceptibility to Helicobacter pylori infection. Helicobacter.

[40] Weixi L., Zhicheng Y., Yan T., Zhang H., Liu R. (2020). Associations of the IL-1B level, IL-1A and IL-1B gene polymorphisms and ankylosing spondylitis risk in a Chinese Han population. Cytokines.

[41] Deirmengian C., Hallab N., Tarabishy A., Della Valle C., Jacobs J.J., Lonner J., Booth R.E. (2010). Synovial fluid biomarkers for periprosthetic infection. Clin. Orthop. Relat. Res..

[42] Glehr M., Friesenbichler J., Hofmann G., Bernhardt G.A., Zacherl M., Avian A., Windhager R., Leithner A. (2013). Novel biomarkers to detect infection in revision hip and knee arthroplasties. Clin. Orthop. Relat. Res..

[43] Milinkovic I., Djinic Krasavcevic A., Nikolic N., Aleksic Z., Carkic J., Jezdic M., Jankovic S., Milasin J. (2021). Notch down-regulation and inflammatory cytokines and RANKL overexpression involvement in peri-implant mucositis and peri-implantitis: A cross-sectional study. Clin. Oral. Implant. Res..

[44] Wang X., Zhang J., Ji J. (2022). IL-1β-induced pentraxin 3 inhibits the proliferation, invasion and cell cycle of trophoblasts in preeclampsia and is suppressed by IL-1β antagonists. Mol. Med. Rep..

