# Supplementary figures and images for: Interleukin-1β Polymorphisms Are Genetic Markers of Susceptibility to Periprosthetic Joint Infection in Total Hip and Knee Arthroplasty
Source: Genes (Basel). 2024 May 8;15(5):596. doi: 10.3390/genes15050596 (PMC11120921; doi:10.3390/genes15050596)

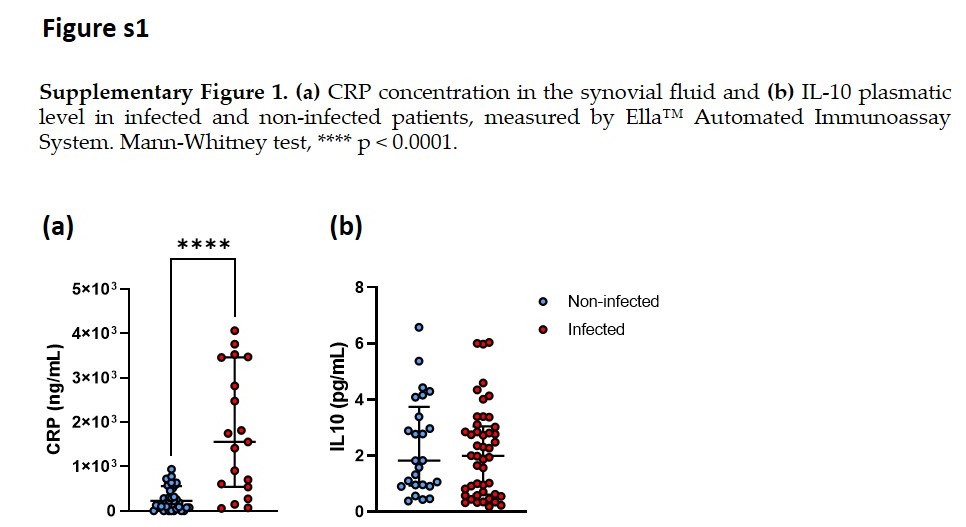

Supplement: Supplementary file 1 [file genes-15-00596-s001.zip › genes-2989880-Supplementary Figure S1.jpg]
